# The biological burden of conflict across populations worldwide

**DOI:** 10.1002/ctm2.70574

**Published:** 2025-12-26

**Authors:** Juan F. Cardona, Hernando Santamaría‐García, Agustín Ibáñez

**Affiliations:** ^1^ Department of Developmental Sciences Cognition and Neuroscience Faculty of Psychology Universidad del Valle Cali Colombia; ^2^ Global Brain Health Institute (GBHI) University of California San Francisco San Francisco California USA; ^3^ Center of Memory and Cognition Intellectus Hospital Universitario San Ignacio Bogotá Colombia; ^4^ PhD Program in Neuroscience Pontificia Universidad Javeriana Bogotá Colombia; ^5^ Latin American Brain Health Institute (BrainLat) Universidad Adolfo Ibañez Santiago de Chile Chile; ^6^ Department of Biophysics, School of Medicine Istanbul Medipol University Istanbul Turkiye; ^7^ Cognitive Neuroscience Center (CNC) Universidad de San Andrés Buenos Aires Argentina; ^8^ Global Brain Health Institute (GBHI) Trinity College Dublin (TCD) Dublin Ireland

Cardona JF, Santamaría‐García H, Ibáñez A. The biological burden of conflict across populations worldwide. *Clin Transl Med*. 2025;00:e70574. https://doi.org/10.1002/ctm2.70574

Contemporary societies are increasingly shaped by multilayered forms of interindividual and intergroup conflict that transcend traditional political or military boundaries.^1^, ^2^ Violence, forced migration, environmental degradation, institutional fragility and widening social and political polarisation interact to generate complex forms of adversity that affect entire populations. These conditions carry profound consequences for mental and brain health, yet their biological impact remains insufficiently integrated into global health and policy agendas. Recent analyses show that even political polarisation itself now functions as a determinant of population health, shaping stress, trust, behaviour and risk perception.^3^ Thus, understanding the biological burden of conflict requires a genuinely transdisciplinary perspective capable of linking neurobiological mechanisms with social determinants, ecological pressures and institutional dynamics.

## 1 CONFLICT EVERYWHERE

**FIGURE 1:**
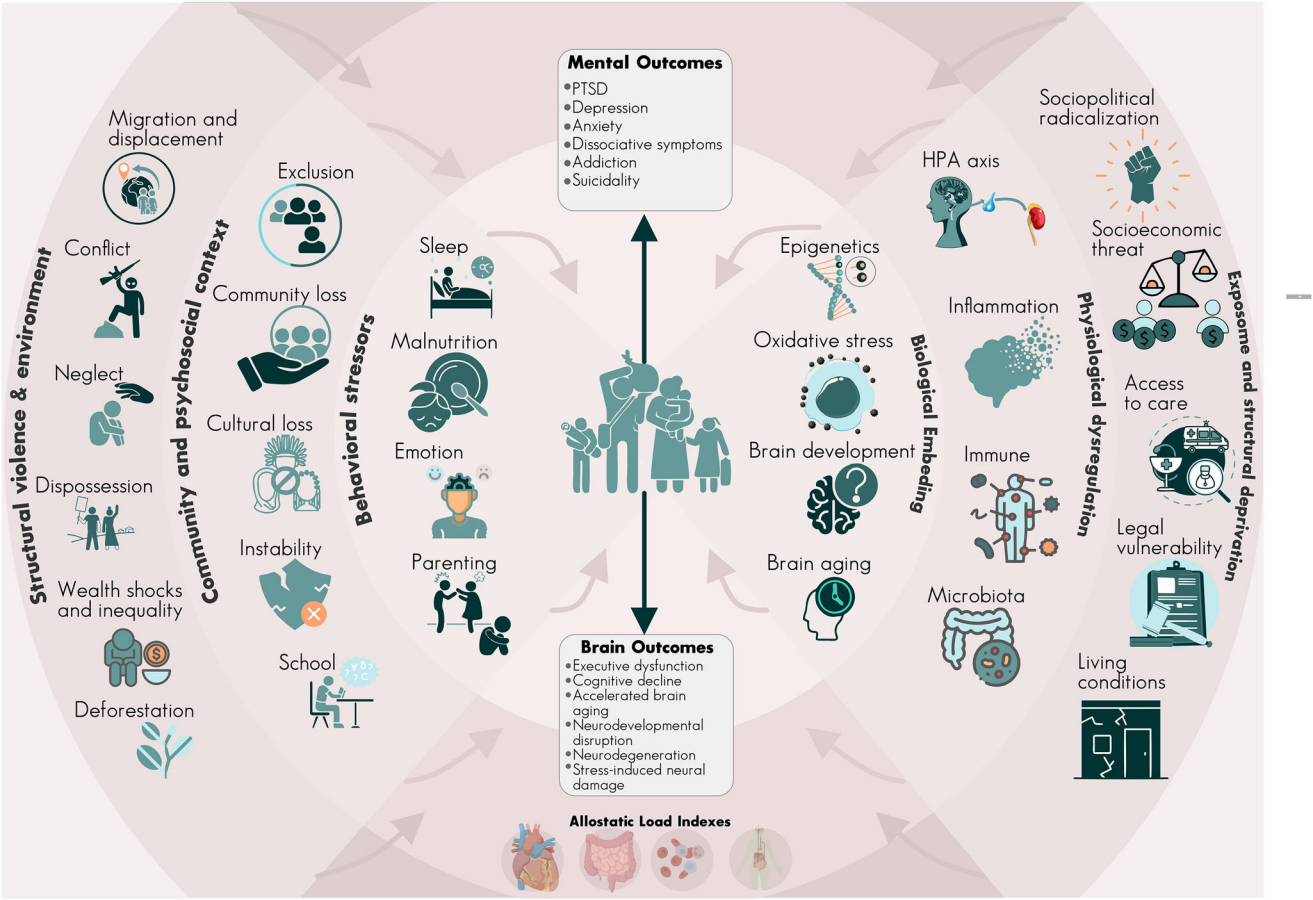
Conflict‐related exposome and multilevel mechanisms of brain and mental health. Conceptual frameworks linking structural violence and social adversity to brain and mental health outcomes via behavioural, physiological and biological pathways. Adversities are organized into three domains: (1) structural and environmental factors (e.g. forced displacement, inequality and dispossession), (2) community and psychosocial stressors (e.g. malnutrition, parental stress and instability), and (3) exposome and biological mediators (e.g. inflammation, HPA axis dysregulation and microbiota). These domains interact through allostatic load mechanisms, leading to mental health outcomes and brain changes. The central figure highlights the cumulative burden of violence and deprivation, illustrating how chronic exposure becomes biologically embedded in conflict‐affected populations.

Although classical armed conflicts persist, today's adversities extend beyond the battlefield. Many societies face recent escalating political polarisation, intergroup conflict, radicalisation, recurrent violence, institutional erosion, widening inequality and expanding illicit economies, including the resurgence of narcotrafficking and environmentally destructive extractive activities. High‐income regions are not exempt; rising polarisation, mass shootings, hate crimes, digital extremism and climate‐related displacement in the United States and Europe illustrate how conflict‐like dynamics increasingly permeate daily life (Figure 1).

Globally, conflict‐related adversity is intensifying. Countries such as Ukraine, Gaza, Sudan, Myanmar, Venezuela, Colombia and those across the Sahel experience interrelated crises involving displacement, food insecurity, environmental loss, organised violence and institutional collapse. As the United Nations recently stressed, ethnic violence and nationalist aggression continue to drive conflicts that displace millions and generate prolonged humanitarian emergencies.^4^ Despite differences in political structure or geography, these contexts converge through shared mechanisms such as chronic adversity, disrupted community structures, cumulative stress exposure and the erosion of protective social and ecological systems.

Colombia provides a clear illustration of how contemporary conflicts can reconfigure themselves across time.^5^, ^6^, ^7^ After the 2016 Peace Agreement, territorial fragmentation, the proliferation of armed groups, the expansion of illegal economies, and widespread narco‐deforestation generated new cycles of violence and displacement. More than one million new displacements since 2016 reflect a persistent humanitarian and mental health burden.^7^ These overlapping crises, environmental degradation, forced migration, urban overcrowding and community fragmentation, highlight how conflict‐related adversity can accumulate across social and ecological systems, creating conditions with direct implications for mental and brain health.

## 2 HOW CONFLICT SHAPES MENTAL, BRAIN AND WHOLE‐BODY HEALTH

Conflict‐related adversity affects health beyond psychological trauma.^8^, ^9^ Chronic exposure to threat, instability, deprivation, and displacement produces sustained physiological stress that alters circadian rhythms, metabolic pathways, cardiovascular regulation and immune functioning.^10^, ^11^ These multisystem disruptions increase vulnerability to psychiatric disorders, cognitive decline, accelerated ageing and long‐term somatic disease.

At the neurobiological level, cumulative adversity triggers allostatic overload marked by HPA‐axis dysregulation, chronic inflammation, immune activation and altered autonomic balance.^12^ These mechanisms impair neuroplasticity and reorganise large‐scale brain networks involved in salience detection, memory, emotional regulation and social cognition. These neural signatures are inseparable from broader whole‐body physiology: accelerated epigenetic ageing, telomere shortening, vascular dysfunction, sleep disruption and alterations in the microbiota–gut–brain axis. The exposome framework provides an integrative lens, showing how physical, social and environmental exposures accumulate and become biologically instantiated across the lifespan.^13^ Conflict, in this sense, operates as a concentrated exposome of structural violence.

These biological alterations translate into well‐documented mental and brain health outcomes, including increased rates of PTSD, depression, anxiety, suicidality, dissociative symptoms, executive dysfunction, emotional dysregulation and accelerated cognitive ageing.^12^, ^14^ Neuroimaging studies align with these findings, showing reduced hippocampal and prefrontal volumes, altered cortical thickness, white‐matter disruptions and reorganised default mode and salience networks in individuals exposed to severe violence or displacement.^15^, ^16^

## 3 WHY A TRANSDISCIPLINARY APPROACH IS ESSENTIAL

Conflict has long been treated as a political or security issue rather than a determinant of brain and mental health. Yet the evidence shows conflict across many manifestations generates multisystem physiological consequences. Biology alone cannot explain patterns of suffering rooted in land dispossession or legal precarity. Social science alone cannot capture neural dysregulation. Public health alone cannot address the ecological and institutional drivers that maintain adversity.

A transdisciplinary framework is indispensable. Integrating mental and brain health with environmental science, justice systems, social protection, and economic policy is essential to understand and mitigate the biological embedding of conflict. Research agendas must connect neuroimaging, psychophysiology, and molecular markers with fine‐grained measures of displacement, violence exposure, land‐use change, pollution, and institutional trust. Trauma‐informed care must be paired with interventions on housing, livelihoods, schooling, and ecosystem restoration. Syndemic theory provides a scaffold for this integration, and the emerging concept of neurosyndemics extends it to the nervous system, highlighting how conflict‐related adversities co‐produce interacting epidemics of mental disorders, neurological disease, and accelerated ageing.^17^

## 4 A TRANSLATIONAL AGENDA FOR GLOBAL CONFLICT AND HEALTH

Mental and brain health should be explicitly integrated into peacebuilding frameworks, transitional justice processes and national recovery agendas. Community‐based, trauma‐informed care must be expanded in marginalised territories. Exposome‐ and One Health–informed strategies are needed to incorporate environmental determinants, deforestation, land degradation, pollution, and biodiversity loss into mental health planning.^17^, ^18^ Finally, multisectoral interventions that pair clinical care with social protection and environmental restoration are required to break feedback loops between conflict, illness, and ecological collapse.

Contemporary conflict is not only a geopolitical or security problem but a chronic, biologically embedded exposure that reshapes stress systems, brain health, and accelerated ageing across generations. Violence, displacement, institutional erosion, and environmental collapse act as a compound exposome of structural violence, driving syndemic epidemics of mental disorders, neurological disease, and accelerated ageing. Addressing this burden demands a transdisciplinary and translational agenda, linking neuroscience with social and environmental sciences, and integrating mental and brain health into peacebuilding and recovery policies to transform conflict from a silent determinant into a central target for global prevention and justice.

## CONFLICT OF INTEREST STATEMENT

The authors declare no conflict of interest.

## FUNDING INFORMATION

Hernando Santamaría‐García is supported by the Davos Alzheimer's collaborative. Agustín Ibáñez is supported by the Multi‐Partner Consortium to Expand Dementia Research in Latin America (ReDLat), supported by the Fogarty International Center (FIC), the National Institutes of Health, the National Institute on Aging (R01 AG057234, R01 AG075775, R01 AG21051, R01 AG083799 and CARDS‐NIH 75N95022C00031), the Alzheimer's Association (SG‐20‐725707), the Rainwater Charitable Foundation – The Bluefield Project to Cure FTD, and the Global Brain Health Institute. Agustín Ibáñez is also supported by ANID/FONDECYT Regular (1250091, 1210176 and 1220995) and ANID/FONDAP/15150012. The contents of this publication are solely the author's responsibility and do not represent the official views of these institutions.
